# Bamboo fiber improves piglet growth performance by regulating the microbial composition of lactating sows and their offspring piglets

**DOI:** 10.3389/fmicb.2024.1411252

**Published:** 2024-07-11

**Authors:** Fawen Dai, Tao Lin, Muqu Jin, Xia Huang, Lu Wang, Jing Ma, Hang Yu, Xianlin Fan, Xiang Nong, Jianjun Zuo

**Affiliations:** ^1^Leshan Normal University, Leshan, China; ^2^Sichuan Provincial Engineering and Technology Research Center for Innovative Development of Bamboo Fiber Nutrition, Leshan, China; ^3^Key Laboratory of Bamboo Pest Control and Resource Development, Leshan, Sichuan, China; ^4^College of Animal Science, South China Agricultural University, Guangzhou, China

**Keywords:** bamboo powder, lactating sows, feed intake, weaning litter weight, fecal microflora

## Abstract

**Introduction:**

Feeding bamboo powder is a kind of fiber raw material mainly composed of insoluble dietary fiber (IDF). In this study, IDF-based rice husk meal and feeding bamboo powder were used to compare the effects of bamboo fiber on fecal microflora and the performance of lactating sows and their offspring piglets.

**Methods:**

Thirty healthy crossbred gilts (Yorkshire × Landrace) at day 105 of gestation were randomly allocated into three groups: CON, TRE1 supplemented with 2% BBF1 (feeding bamboo powder), and TRE2 supplemented with 2% BBF2 (99% feeding bamboo powder +1% bamboo fiber polymer material). The reproductive performance, serum indexes, and fecal microbiota of sows and piglets were analyzed. The results showed that, compared with CON, the average feed intake of sows in TRE1 during the second week of lactation was significantly increased by 21.96% (*p* < 0.05), the average daily gain (ADG) per litter in TRE1 on 11–21 days and 3–21 days of lactation was significantly increased by 50.68 and 31.61%, respectively (*p* < 0.05), and the serum triglyceride content of sows in TRE1 on the 21st day of lactation was significantly increased (*p* < 0.05). The 16S rRNA analysis showed that dietary bamboo fiber significantly increased the fecal microbial richness index Ace, Chao, and Sobs of sows (*p* < 0.05) and tended to increase the Sobs index of suckling piglets on day 21 (*p* < 0.10). Compared with CON, BBF1 supplementation significantly decreased the abundance of *Christensenellaceae_R-7_group* in feces of sows on days 7 and 21 after delivery (*p* < 0.05), while BBF2 decreased the genera *Christensenellaceae_R-7_group* on days 7 (*p* < 0.10) and 21 (*p* < 0.05) after delivery. Spearman correlation analysis showed that the abundance of *Phascolarctobacterium* in the feces of piglets on the 21st day after delivery was significantly positively correlated with diarrhea rate and significantly negatively correlated with ADG per litter, day 21 litter weight, and 3- to 21-day survival rate. In contrast, *Christensenellaceae_R-7_group* was significantly negatively correlated with diarrhea rate and positively correlated with ADG per litter.

**Discussion:**

These results indicated that maternal BBF1 supplementation improved the litter weight gain of suckling piglets, which was associated with the improvement of diversity and structure of the fecal microbiota in the piglets.

## Introduction

Nutritional regulation during pregnancy and lactation is a key factor for the growth and development of their offspring (Zhang et al., 2018), which has a direct impact on the production efficiency and economic benefits of the pig industry. This “sow-piglet” association may be influenced by sow nutrition, which may alter the electrophysiological properties, barrier function, and microbiota of the intestine in their piglets (Awad et al., 2013; Paßlack et al., 2015). The “sows to piglets” model has been manifested in the effect of dietary fiber from different sources, which can regulate the gut microbiota, improve performance, and alleviate inflammatory responses in sows and piglets (Liu et al., 2021). Susceptibility to colonization by *C. difficile* in neonatal piglets can be modulated by the dietary fiber of sows, supporting the hypothesis of early microbial programming in the offspring and the importance of the sow-piglet couple (Grześkowiak et al., 2022). Dietary fiber nutrition for sows may be a better method to improve the performance of their offspring piglets by regulating the microbial composition, which is the main reason for this study.

Different sources of fibers may have different physical and chemical properties, which may cause changes in the composition of the intestinal microbiota (Pi et al., 2021; Shang et al., 2021). Guar gum (soluble dietary fiber, SDF) and cellulose (insoluble dietary fiber, IDF) interacted to increase ileal bifidobacteria and enterobacteria in grower pigs; however, guar gum, but not cellulose, increased ileal clostridia (Owusu-Asiedu et al., 2006). Dietary konjac flour rich in SDF increased the relative abundances of *Akkermansia* and *Roseburia*; however, it had no effect on the α-diversity values of microbial communities (Tan et al., 2016). Compared with coarse wheat bran, dietary supplementation with fine wheat bran with higher SDF significantly increased the Chao index, and the abundance of *Firmicutes* and *Terrisporobacter* was significantly decreased while *Bacteroidetes* and *Parabacteroides* were significantly increased (Wang et al., 2022). Dietary IDF (lignocellulose) significantly increased Lactobacillus amounts in the ileal digesta of piglets, and it was more effective than SDF (insulin) (Chen et al., 2019). These studies suggested that dietary fiber type (SDF and IDF) affected microbial diversity and composition, but the results were inconsistent. Future research needs to gain more insight into the combined effects of SDF and IDF, processing methods, and additional timing to improve the nutritional value of dietary fiber (Li et al., 2021). In addition, increasing the dietary fiber level of sows increased the ileal flow of most nutrients and the total excretion of fecal materials (Serena et al., 2008). Increasing the dietary fiber level of sows in late gestation reversed inflammatory responses and adverse effects by increasing the abundance of Lactobacillus (Li et al., 2023).

China is rich in bamboo species, and the majority are distributed in the southern area (Zhang et al., 2022), especially in the location of this study, which is the hometown of pandas. Young bamboo culms present potential application in the food industry as a source of fibers, with high IDF content accounting for 62.54–89.79% (Felisberto et al., 2017), which was higher than wheat bran, corn bran, and rice bran (Bai et al., 2022). The health-promoting effects of bamboo have been extensively studied in recent years. It has been shown that IDF from bamboo could alter the composition and microbial diversity of gut microbiota, especially by increasing the relative abundance of *Bacteroides* and decreasing the ratio of *Firmicutes* to *Bacteroidetes* (Ge et al., 2022). O-acetylated xylan obtained from bamboo effectively relieved loperamide-induced constipation in mice by significantly improving cecum microbiota composition and shortening defecation time (Huang J. et al., 2022; Huang Y. et al., 2022). Bamboo extracts showed several biological functions, such as anti-inflammatory and antioxidant properties (Tundis et al., 2023). The intake of fermented bamboo fiber improved the immunity, inflammation, and intestinal microbiota of sows, which were closely related to sow performance (Sun et al., 2023). Our previous research has found that micronized bamboo powder can reduce the amount of *Escherichia coli* in the feces of weaned piglets and improve growth performance (Dai et al., 2021), improve cecal microbial composition and increase average daily gain in broilers (Dai et al., 2022), increase the water content in the feces of sows, reduce the oxidative damage, and tend to reduce the relative abundance of opportunistic pathogenic *Fusobacterium* for suckling piglets with a supplement for perinatal sows (Dai et al., 2023). This study was conducted to further study the effects of maternal bamboo fiber supplementation from day 105 of gestation to day 21 post-farrowing on the fecal microbial composition of lactating sows and their piglets on the 1st, 7th, and 21st days after delivery. Reproduction performance, serum biochemical parameters of sows, and growth performance of the piglets were determined.

## Materials and methods

### Test materials

The rice husk meal was crushed over 80 mesh, and the crude fiber was determined to be 31.5% by the AOAC 978.10 method.

Bamboo fiber 1 (BBF1): after removing the bamboo green from the bamboo sticks at 5–6 years, the coarse powder was processed by a cutter mill, dried to <12% moisture, and crushed by a hammer mill over 80 mesh. The content of crude fiber was determined to be 42.7%.

Bamboo Fiber 2 (BBF2): BBF2 was composed of BBF1 and bamboo fiber polymer material (provided by Key Laboratory of Bamboo Pest Control and Resource Development, China) with a ratio of 99:1. The bamboo fiber polymer material was prepared by irradiation treatment and coupling polymer hydrophilic material from *Bambusa emeiensis* at 1 year old. The soluble fiber and total dietary fiber of bamboo fiber were determined to be 19.18 and 78.18%, respectively, by the AOAC 991.43 method, and the crude fiber was determined to be 42.3%.

### Test grouping and treatment

Thirty healthy primiparous sows (Yorkshire × Landrace) with a similar fat condition and the same batch of breeding at day 105 of gestation were randomly allocated into three groups (with 10 replicates per group and 1 pig per replicate): CON (a basal diet group supplemented with 2% rice husk meal), TRE 1 supplemented with 2% BBF1, and TRE 2 supplemented with 2% BBF2. The rice husk meal, BBF1, and BBF2 were directly added to the top access door of a double shaft counterpoise ribbon mixer (hand-adds) with a 1,000-kg full load capacity (SJHS2, Muyang Group, China). The experimental diet was formulated according to the NRC (2012) and GB/T 39235–2020 nutritional requirements of sows. The composition and nutritional levels of experimental diets are shown in Table 1, and the additional level of bamboo fiber was referred to the reports of similar fiber sources (Grześkowiak et al., 2022; Dai et al., 2023; Sun et al., 2023).

**Table 1:** Ingredients and chemical composition of diets (air-dried).

Items	Groups		
	CON	TRE-1	TRE-2
Corn	575.5	575.5	575.5
Soybean meal	215	215	215
Fermentation SBM	24	24	24
Super steamed fish meal	18	18	18
Chili meal	50	50	50
Wheat meal	40	40	40
Rice husk meal	20		
BBF1		20	
BBF2			20
Soybean oil	10	10	10
Limestone	9.5	9.5	9.5
Hydrogen calcium	10.8	10.8	10.8
Lysine 98%	3	3	3
NaCl	4.2	4.2	4.2
Premix^a^	20	20	20
Toal	1,000	1,000	1,000
**Nutrient level**^b^			
DE/(MJ/kg)	13.63	13.63	13.63
NE/(MJ/kg)	9.80	9.80	9.80
CP, %	18.13	18.12	18.12
CF, %	5.12	5.34	5.34
NDF, %	13.94	14.53	14.53
Lys, %	1.15	1.15	1.15
Met, %	0.29	0.29	0.29
Met + Cys, %	0.48	0.48	0.48
Thr, %	0.68	0.68	0.68
Trp, %	0.20	0.20	0.20
Val, %	0.83	0.83	0.83

a Premix provides the following per kilogram of diets: vitamin A, 12,000 IU, VitD_3_ 2,000 IU, VitE 75 mg, VitK_3_ 4.5 mg, VitB_1_ 2.9 mg, VitB_2_ 10 mg, VitB_6_ 3.1 mg, VitB_12_ 0.04 mg, VitC 100 mg, pantothenic acid 26 mg, nicotinic acid 31 mg, folic acid 3.1 mg, choline 600 mg, iron 120 mg, copper 15 mg, zinc 70 mg, manganese 65 mg, iodine 0.4 mg, cobalt 0.2 mg, selenium 0.3 mg, chromium 0.2 mg.

b Nutrient level was the calculated value.

### Feeding management

The experiment was conducted in the experimental pig farm of Wenzhou Zhugeliang Animal Husbandry Co., Ltd., Wenzhou, Zhejiang Province, China, from July to August 2022. Kept in the parturition pen, the experimental sows were fed 3 kg per sow per day before parturition, 1 kg/day on the day of parturition, and 2 kg/day on day 2, then 0.5 kg/day more feed each day from day 3 to day 7, free feeding from day 8 to day 21, and the leftovers were collected and weighed every day. Sows were fed two meals a week before and after delivery at 6:30 and 17:30, respectively. Sows were fed three meals on the first day of the second week after delivery at 5:30, 10:30, and 17:30, respectively. All sows and piglets drank freely, and the environmental conditions in the piggery were consistent. The rest of the feeding management shall be carried out according to the unified procedures of the pig farm.

The pre-feeding period was 2 days, and the formal experiment lasted until the end of weaning at 21 days after delivery. Within 48 h after delivery, the number of piglets carried by each sow was adjusted in the treatment group according to the milk of sows through foster care, consolidation, and other means. In the subsequent experiment, piglets were no longer adjusted, the daily feed intake of sows was accurately recorded, and the feeding amount was increased according to the feeding amount and feeding speed of the previous day (the feeding increase rate did not exceed 0.5 kg/day).

### Sample collection

Fiber feed samples: 500 g rice husk meal, bamboo fiber 1, and bamboo fiber 2 were collected and frozen at −20°C for testing.

Serum samples of sows: six sows in each group were randomly selected on the morning of the 3rd and 21st days (weaning) after delivery, and 5 mL of blood was collected from the ear vein of each sow before meals and placed in a centrifuge tube for 30 min. Serum was separated by centrifuging at 3,000 rpm for 15 min. The serum was absorbed, divided into a 1.5-ml centrifuge tube, and frozen at −20°C for testing.

Fecal samples: on the morning of the 1st, 7th, and 21st days after delivery, six sows in each group were randomly selected. About 300 mg of fecal samples were scraped from inside the stool and placed into a 1.5-ml centrifuge tube. Meanwhile, about 300 mg of fresh fecal samples of piglets born in sows were collected and placed into a 1.5-ml centrifuge tube, frozen with liquid nitrogen, and frozen at −80°C for microbial analysis.

### Reproductive performance of sows

The backfat thickness of sows at point P2 (6 cm from the last dorsal mid-line of ribs on the left side) was measured with the ultrasonic backfat meter (Renco, United States) at the start of the experiment and at weaning time to calculate the backfat loss of sows during lactation. The duration of farrowing, total litter size, healthy litter size (weight over 1 kg), live litter size, litter size of sows in foster adjustment, daily feed intake during lactation, and number of weaned piglets were recorded. Calving intervals, feed intake of sows during the first, second, and third weeks of lactation, and throughout lactation were calculated. Accurately record the birth time of the first piglet and the birth time of the last piglet, and the interval between the two is the labor process.

Average litter interval = length of labor/total litter size.

### Growth performance of suckling piglets

The number of piglets, the number of piglets with diarrhea, and the number of stillborn piglets were recorded every day in the unit of repetition, and the weights of piglets at birth, day 3, day 10, and day 21 were weighed by litters. Average birth weight, average individual weight on days 3, 10, and 21, average daily gain of litters on days 3–11, days 11–21, and days 3–21, average daily gain of individuals on days 3–11, days 11–21, and days 3–21, and diarrhea rate of piglets on days 3–11, days 11–21, and days 3–21 were calculated. The survival rate of piglets on days 3–11, days 11–21, and days 3–21 was calculated.

Average birth weight (kg) = litter weight/number of live offspring.

Average body weight (kg) = litter weight/number of piglets.

Average daily gain (ADG) per litter (kg/d) = (litter weight at time point 2- litter weight at time point 1)/interval days.

Individual average daily gain (g/d) = (body weight at time point 2- body weight at time point 1)/interval days.

Diarrhea rate of piglets (%) = (Number of piglets with diarrhea × days of diarrhea)/(total number of piglets × days of feeding) × 100%.

Piglet survival rate (%) = Number of piglets at time point 2 / Number of piglets at time point 1 × 100%.

### Determination of serum biochemical indexes

The serum biochemical indexes of sows included triglyceride (TG), total cholesterol (T-CHO), total protein (TP), and urea nitrogen (BUN), all of which were determined by the kits purchased from Nanjing Jiancheng Institute of Bioengineering (Jiangsu, China). Serum TG, T-CHO, and BUN were determined by the enzymatic method with the kit numbers A110-1-1, A111-1-1, and C013-2-1, respectively. Serum TP was determined by the ceruloplasmin method with kit number A029.

### Fecal microbial analysis

DNA extraction kit (Omega Bio-tek, Norcross, GA, United States) was used to extract genomic DNA from stool samples of experimental sows and piglets, and 1% agarose gel electrophoresis was used to detect the quality of the extracted genomic DNA. DNA concentration and purity were determined using NanoDrop2000 (Thermo Scientific Inc., United States). Taking that DNA as a template, PCR amplification of the V3–V4 variable region of the 16S rRNA gene was performed using priors 338F (5′-ACTCCTACGGGAGGCAGCAG-3′) and 806R (5′-GGACTACHVGGGTWTCTAAT-3′) (Liu et al., 2016). Paired-end sequencing of 16S rRNA PCR products was performed on the Illumina MiSeq PE300 platform by Shanghai Meiji Biomedical Technology Co., Ltd.

Fastp software^1^ was applied for quality control of double-ended original sequencing sequences (Chen et al., 2018). FLASH software^2^ was used for stitching (Magoč and Salzberg, 2011). (1) The base of reads with a mass value below 20 at the tail end was filtered, and a 50-bp window was set. If the average mass value in the window was lower than 20, the back-end bases were cut off from the window. Reads below 50 bp after quality control were filtered, and reads containing N-base were removed. (2) According to the overlap between PE reads, pairs of reads were merged into a sequence with a minimum overlap length of 10 bp. (3) The maximum mismatch ratio of the overlap region of the spliced sequences was 0.2, and the inconsistent sequences were screened. (4) The samples were differentiated according to the barcode and primers at both ends of the sequence, and the sequence direction was adjusted. The allowable mismatch number of barcode was 0, and the maximum mismatch number of primer was 2.

Using UPARSE software^3^ (Stackebrandt and Goebel, 1994; Edgar, 2013), the operational taxonomic unit (OTU) was used to cluster the quality control concatenated sequences and eliminate chimeras based on 97% similarity. In order to minimize the impact of sequencing depth on subsequent alpha and beta diversity data analysis, the sequence number of all samples was reduced to 34,060, and the average sequence coverage of each sample could still reach 99.49% after being reduced. OTU species taxonomy annotation was performed by comparing the RDP classifier^4^ with the Silva 16S rRNA gene database (Release138^5^) with a confidence threshold of 70%. The community composition of each sample was counted at different species classification levels (Wang et al., 2007).

### Data sorting and statistical analysis

One-way ANOVA program of SPSS 23.0 statistical software was used to conduct a one-way analysis of variance. Reproductive performance, growth performance, and serum indexes were statistically analyzed with the LSD method for multiple comparisons and an independent-sample *t*-test for the difference between means. The results were expressed as mean ± standard error; *p* < 0.05 indicated significant differences, and 0.05 ≤ *p* < 0.10 indicated a difference trend.

The 16S rRNA sequencing analysis of all samples was performed on the Meggie Biocloud platform^6^ as follows: alpha diversity, including the Ace, Chao, and Sobs, richness index, Shannon and Simpson index characterization of diversity, is obtained by using the mothur software.^7^ The Wilcoxon rank sum test was used to analyze the difference in alpha diversity between groups. Principal coordinate analysis (PCoA) based on the Bray-Curtis distance algorithm was used to test the similarity of microbial community structure among samples. ANOSIM non-parametric test was combined to analyze whether the differences in microbial community structure between samples were significant. The Student’s *t*-test was used to analyze the differences in the relative abundance of major microorganisms at phylum and genus levels between different experimental groups. *p* < 0.05 indicated a significant difference, and 0.05 ≤ *p* < 0.10 indicated a trend of difference.

## Results

### Effects of maternal bamboo fiber supplementation on reproductive performance

As seen from Table 2, the average daily feed intake (ADFI) of sows fed with bamboo fiber 1 in the second week after delivery was significantly higher in TRE1 than that in the rice husk meal CON (*p* < 0.05), increased by 21.96%, and increased by 7.67, 9.54, and 13.08% in the first week, third week, and whole lactation, respectively, but did not reach the significant difference level (*p* > 0.05). Bamboo fiber treatment had no significant effects on farrowing duration, farrowing interval, litter size, healthy litter size, back fat thickness at weaning, or back fat loss during the trial period. These results indicated that the dietary addition of 2% bamboo fiber 1 can improve the feed intake of sows during lactation, mainly in the mid-lactation (the second week after delivery).

**Table 2:** Effects of BBF on the reproductive performance of sows.

Items	CON	TRE1	TRE2	SEM	*p*-value
Labor, min	281.71	249.22	221.33	35.50	0.822
Farrowing interval, min	14.15	17.62	17.11	2.01	0.778
**Litter size**					
Total litter size	14.86	15.67	13.71	0.50	0.279
Total born alive litter size	14.71	15.00	13.86	0.40	0.507
Healthy litter size	12.29	12.00	11.43	0.64	0.879
After cross-foster	13.71	14.00	13.57	0.17	0.566
Weaning litter size	11.29	13.00	11.00	0.45	0.121
**Average daily feed intake, kg/day**					
Week 1	2.87	3.09	2.90	0.12	0.734
Week 2	3.37^a^	4.11^b^	3.73^ab^	0.12	0.037
Week 3	4.09	4.48	4.16	0.16	0.560
Weeks 1–3	3.44	3.89	3.59	0.12	0.275
**Backfat thickness, mm**					
Initial backfat thickness	19.86	20.67	21.14	0.69	0.778
Weaning backfat thickness	16.43	16.67	16.00	0.48	0.860
Backfat thickness change	3.43	4.00	5.14	0.40	0.234

*n* = 10 for each group, p < 0.05 means a significant difference.

^a,b^Means in the same row, different superscript letters show significant differences (*p* < 0.05).

### Effects of maternal bamboo fiber supplementation on growth performance of piglets

As shown in Table 3, dietary supplementation of bamboo fiber in sows had a significant effect on the average daily gain (ADG) per litter in piglets at pre-weaning 11–21 days and 3–21 days (*p* < 0.05), and ADG per litter in TRE1 supplemented with bamboo fiber 1 was significantly higher than that in CON or that in TRE2 group (*p* < 0.05), increasing by 50.68 and 51.70%, 31.61 and 34.71%, respectively. The 21-day weaning body weight (BW) per litter of TRE1 was significantly higher than that of TRE2 group (*p* < 0.05) and tended to be higher than that of CON (*p* < 0.10). The survival rate of 3- to 21-day-old piglets in TRE1 was higher than that in CON group and TRE2 group, which increased by 13.70% (*p* < 0.10) and 14.92% (*p* < 0.10), respectively. These results indicated that dietary supplementation of 2% bamboo fiber 1 in sows can improve the performance of piglets, mainly in the survival rate of piglets, weaning litter weight, and ADG per litter.

**Table 3:** Effects of BBF on the growth performance of piglets.

Items	CON	TRE1	TRE2	SEM	*p*-value
**Litter weight, kg**					
Born litter weight	19.06	18.74	16.68	0.58	0.223
3rd-day litter weight	22.80	22.63	22.19	0.97	0.970
10th-day litter weight	39.26	41.65	38.17	1.44	0.604
21st-day litter weight	54.10^ab^	63.93^b^	52.84^a^	2.24	0.065
**Mean body weight, kg**					
Born body weight	1.31	1.26	1.18	0.04	0.493
3rd-day body weight	1.65	1.61	1.63	0.06	0.958
10th-day body weight	3.00	3.04	3.04	0.07	0.972
21st-day body weight	4.77	4.93	4.85	0.08	0.709
**Average daily gain per litter, kg/day**					
3–10 days	2.06	2.38	2.00	0.10	0.199
11–21 days	1.48^a^	2.23^b^	1.47^a^	0.13	0.009
3–21 days	1.74^a^	2.29^b^	1.70^a^	0.10	0.012
**Average daily gain per piglet, g/day**					
3–10 days	168.98	179.14	170.53	3.74	0.487
11–21 days	176.63	188.90	180.67	8.52	0.840
3–21 days	173.23	184.56	178.88	4.96	0.659
**Diarrhea rate, %**					
3–10 days	1.96	1.43	0.91	0.25	0.269
10–21 days	2.26	1.86	3.61	0.59	0.472
3–21 days	2.12	1.66	2.33	0.34	0.719
**Survival rate, %**					
3–10 days	93.55	97.55	92.76	1.44	0.338
10–21 days	86.76	95.13	87.43	2.28	0.235
3–21 days	81.70	92.89	80.83	2.74	0.113

*n* = 10 for each group, *p* < 0.05 means a significant difference.

^a,b^Means in the same row, different superscript letters show significant differences (*p* < 0.05).

### Effects of maternal bamboo fiber supplementation on serum biochemical indices of sows

As can be seen from Table 4, on the 21st day after delivery, the serum triglyceride content of sows in TRE1 and TRE2 was significantly higher than that in rice husk meal CON (*p* < 0.05), and the total cholesterol content was higher than that in CON, but the difference was not significant (*p* > 0.05). On the third day after delivery, the total cholesterol and total triglyceride contents of sows in TRE1 were higher than those in CON, but the difference was not significant (*p* > 0.05). Bamboo fiber treatment had no significant effects on serum total protein or urea nitrogen. The above results indicated that dietary supplementation of 2% bamboo fiber can promote the lipid metabolism of sows in the late lactation period.

**Table 4:** Effects of BBF on serum biochemical parameters of sows.

Items	CON	TRE1	TRE2	SEM	*p*-value
**3rd day**					
TC, mmol/L	1.45	4.42	3.03	0.79	0.320
TG, mmol/L	0.81	1.20	0.86	0.10	0.210
TP, mg/ml	81.07	67.99	61.64	6.47	0.490
UN, mg/L	29.95	31.30	28.00	0.74	0.200
**21st day**					
TC, mmol/L	1.73	2.68	2.24	0.24	0.310
TG, mmol/L	0.48^a^	1.72^b^	1.33^b^	0.19	0.010
TP, mg/ml	62.13	47.07	46.74	3.94	0.200
UN, mg/L	30.32	29.03	34.42	1.46	0.310

TC, total cholesterol; TG, total triglycerides; TP, total protein; UN, urea nitrogen.

*n* = 6 for each group, *p* < 0.05 means a significant difference.

^a,b^Means in the same row, different superscript letters show significant differences (*p* < 0.05).

### Effects of maternal bamboo fiber supplementation on fecal microbial sequence data and alpha and beta diversity of sows and suckling piglets

**Figure 1:**
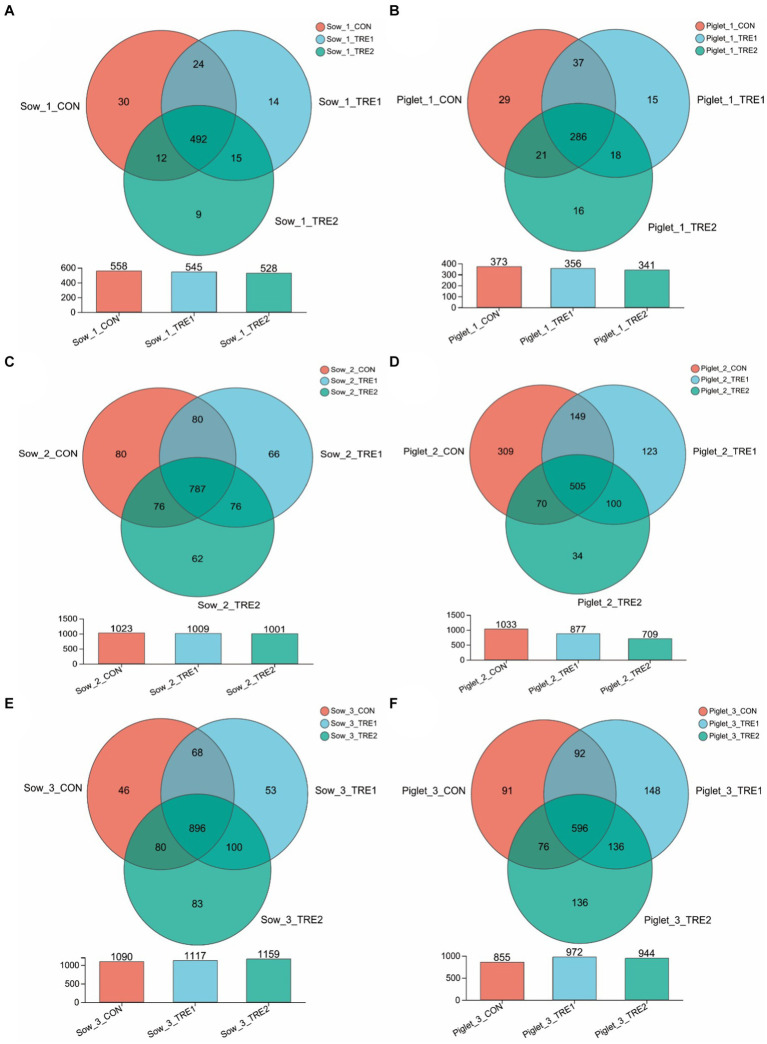
OTU Vane diagram of fecal microorganisms of sows and suckling piglets in different experimental groups. **(A,C,E)** Show sows on the first day after delivery, the seventh day after delivery, and the 21st day after delivery, respectively. **(B,D,F)** Show piglets on the first day after delivery, the seventh day after delivery, and the 21st day after delivery, respectively. Sow_1_CON, Sow_1_TRE1, and Sow_1_TRE2, Piglet_1_CON, Piglet _1_TRE1, and Piglet _1_TRE2 represented sows in CON, TRE1, and TRE2, suckling piglets in CON, TRE1, and TRE2 on the first day after delivery, respectively. Sow_2_CON, Sow_2_TRE1, and Sow_2_TRE2, Piglet_2_CON, Piglet _2_TRE1, and Piglet _2_TRE2 represented sows in CON, TRE1, and TRE2, suckling piglets in CON, TRE1, and TRE2 on the seventh day after delivery, respectively. Sow_3_CON, Sow_3_TRE1, and Sow_3_TRE2, Piglet_3_CON, Piglet _3_TRE1, and Piglet _3_TRE2 represented sows in CON, TRE1, and TRE2, suckling piglets in CON, TRE1, and TRE2 on the 21st day after delivery, respectively. *n* = 6 for each group. Same in Figures 2–6.

To investigate the effects of bamboo fiber on fecal microbial diversity and abundance of sows and suckling piglets at different postpartum stages, PCR amplification and Illumina MiSeq high-throughput sequencing were performed on the 16S rRNA V3–V4 region of bacteria in each sample. According to the statistics of OTU after drawing out, it can be seen from Figure 1 that the number of fecal microbial OTU of sows and piglets on the day of delivery was lower than those on the 7th and 21st days after delivery, and the differences in OTU numbers between TRE1 and TRE2 were greater on the 7th and 21st days after delivery. On the day of delivery, the fecal OTUs of sows in CON were 558, and the fecal OTUs of sows in TRE1 and TRE2 were 545 and 528, respectively, which were lower than those in CON. Compared with CON, the unique OTU numbers of sows in TRE1 and TRE2 were 29 and 24, respectively. The numbers of piglets fecal OTU in TRE1 and TRE2 were 356 and 341, respectively, which were lower than those in CON (373). Compared with CON, the unique fecal OTUs were 33 and 34 in TRE1 and TRE2, respectively.

On the seventh day after delivery, the fecal OTU of sows in CON was 1,023, and the fecal OTU of sows in TRE1 and TRE2 were lower than those in CON (1,009 and 1,001, respectively). Compared with CON, the unique OTUs of sows in TRE1 and TRE2 were 138 and 142, respectively. The fecal OTUs in TRE1 and TRE2 were 877 and 709, respectively, which were lower than those in CON (1033). Compared with CON, the unique fecal OTUs were 134 and 223, respectively, in TRE1 and TRE2. On the 21st day after delivery, the fecal OTUs of sows in CON were 1,090, and the fecal OTUs of sows in TRE1 and TRE2 were higher than those in CON (1,117 and 1,159, respectively). Compared with CON, the unique OTUs of sows in TRE1 and TRE2 were 153 and 183, respectively. The fecal OTUs in TRE1 and TRE2 were 972 and 944, respectively, which were lower than those in CON (855). Compared with CON, there were 284 and 272 unique fecal OTUs in TRE1 and TRE2, respectively. These results indicated that bamboo fiber had a different effect on the quantity of fecal microbe OTU in sows than chaff, and the effects on sows and suckling piglets in late lactation were more obvious than those in early lactation.

Bacterial alpha diversity indexes were shown in Table 5, sows fecal microbial representation richness index Ace, Chao, and Sobs were significantly increased by dietary bamboo fiber (*p* < 0.05) and tended to increase the Sobs index of suckling piglets on day 21 (*p* < 0.10). Maternal bamboo fiber supplementation had no effects on Ace, Chao, Shannon, and Simpson indexes in suckling piglets (*p* > 0.05). Fecal Ace, Chao, Sobs, Shannon, and Simpson indexes were significantly increased by the reproductive ages in sows and suckling piglets (*p* < 0.05) but not affected by the diet × age interaction (*p* > 0.05).

**Table 5:** The alpha diversity in each group of sows and piglets.

Item	L1			L7			L21			SEM	*p*-values		
	CON	TRE1	TRE2	CON	TRE1	TRE2	CON	TRE1	TRE2		Diet	Stage	Diet × stage
**Sow**													
Sobs	335.67	389.00	389.17	539.67	568.17	565.83	591.67	654.50	676.83	122.68	0.000	0.000	0.504
Ace	398.17	437.18	440.13	684.55	723.71	726.71	727.90	800.97	845.18	166.48	0.000	0.000	0.184
Chao	405.21	445.76	440.61	692.50	724.88	747.68	730.04	810.62	849.77	169.20	0.000	0.000	0.295
Shannon	2.90	3.51	3.34	3.20	3.05	3.08	3.94	4.03	4.11	0.62	0.442	0.000	0.355
Simpson	0.21	0.11	0.13	0.15	0.18	0.16	0.06	0.08	0.06	0.09	0.690	0.001	0.272
**Piglet**													
Sobs	203.67	195.33	186.67	365	407.67	282.5	358	423.5	392.83	114.1	0.087	0.000	0.182
Ace	234.2	229.21	217.67	475.91	501.49	384.16	484.48	543.16	525.13	153.77	0.265	0.000	0.355
Chao	234.82	232.97	226.98	471.47	499.79	393.32	470.85	550.02	520.14	155.42	0.232	0.000	0.297
Shannon	3.21	3.16	3.28	3.41	3.44	3.35	3.82	3.95	3.7	0.46	0.871	0.000	0.869
Simpson	0.11	0.1	0.08	0.09	0.1	0.08	0.05	0.05	0.07	0.05	0.952	0.020	0.542

Sows and piglets were regarded as the experimental units, *n* = 6 for each group. L1: day 1 of lactation; L7: day 7 of lactation; L21: day 21 of lactation. When significant main effects or interactive effects were observed, the means were compared using the least significant difference method with a *p* < 0.05 indicating significance.

**Figure 2:**
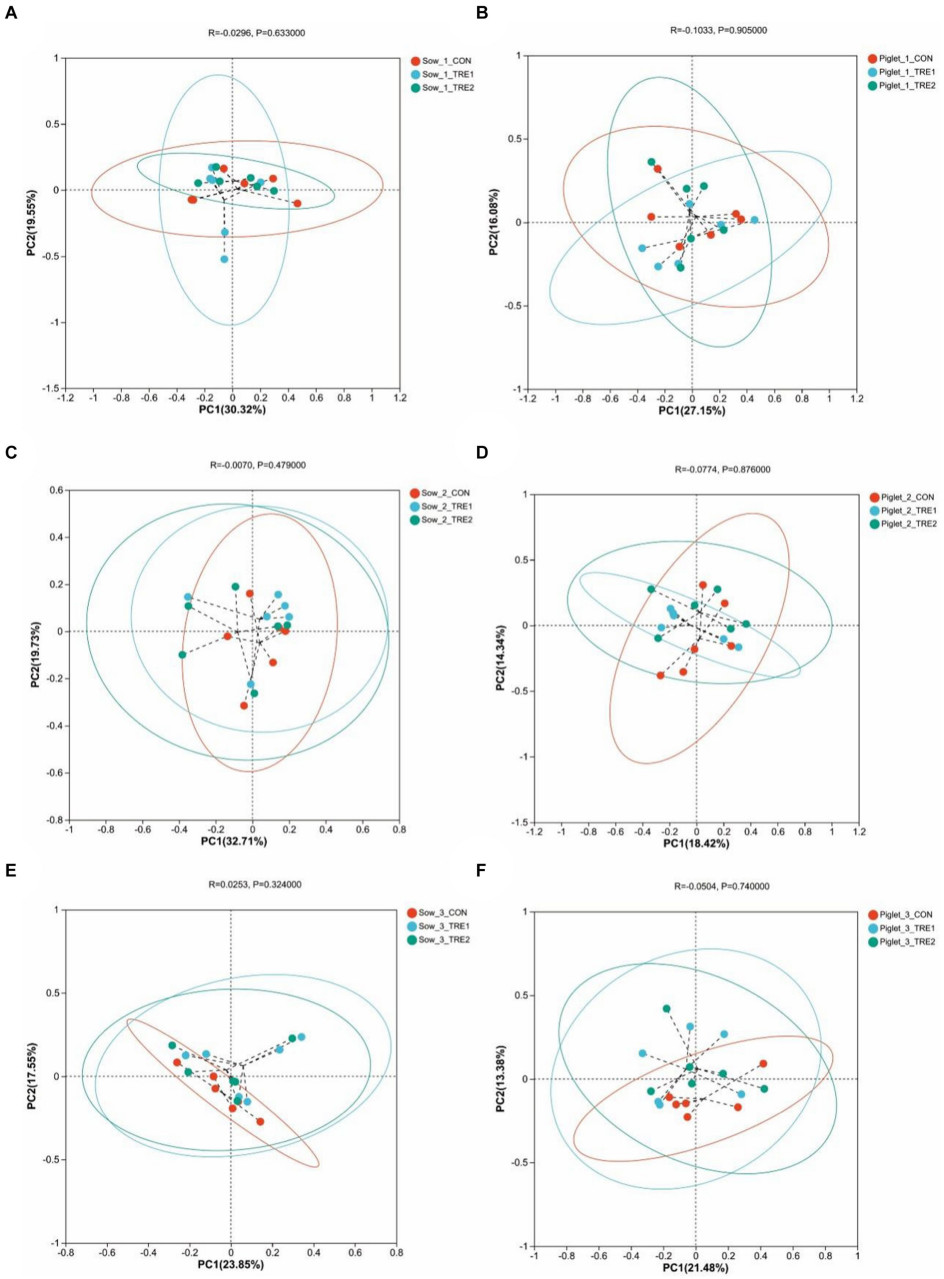
Beta-diversity analysis among experimental groups. Principal coordinates analysis (PCoA) for sows in the control group (CON), treatment group 1 (TRE1), and treatment group 2 (TRE2) on the first day after delivery **(A)**, the seventh day after delivery **(C)**, and the 21st day after delivery **(E)**. Principal coordinates analysis (PCoA) for suckling piglets in the control group (CON), treatment group 1 (TRE1), and treatment group 2 (TRE2) on the first day after delivery **(B)**, the seventh day after delivery **(D)**, and the 21st day after delivery **(F)**. *n* = 6 for each group.

To investigate the effects of supplementary bamboo fiber on fecal microbial beta diversity, PCoA was used to determine the differences among treatment groups. Principal coordinate analysis was conducted based on the Bray-Curtis distance of relative abundance of fecal microbial colonies in the OTU of sows and their suckling piglets. As shown in Figure 2, the fecal microbiota structures of sows in TRE1 and TRE2 were similar to those in CON on the 1st, 7th, and 21st days after delivery, and the fecal microbiota structures of their suckling piglets in different treatment groups were also similar.

### Effects of maternal bamboo fiber supplementation on the fecal microbial composition of sows and suckling piglets

**Figure 3:**
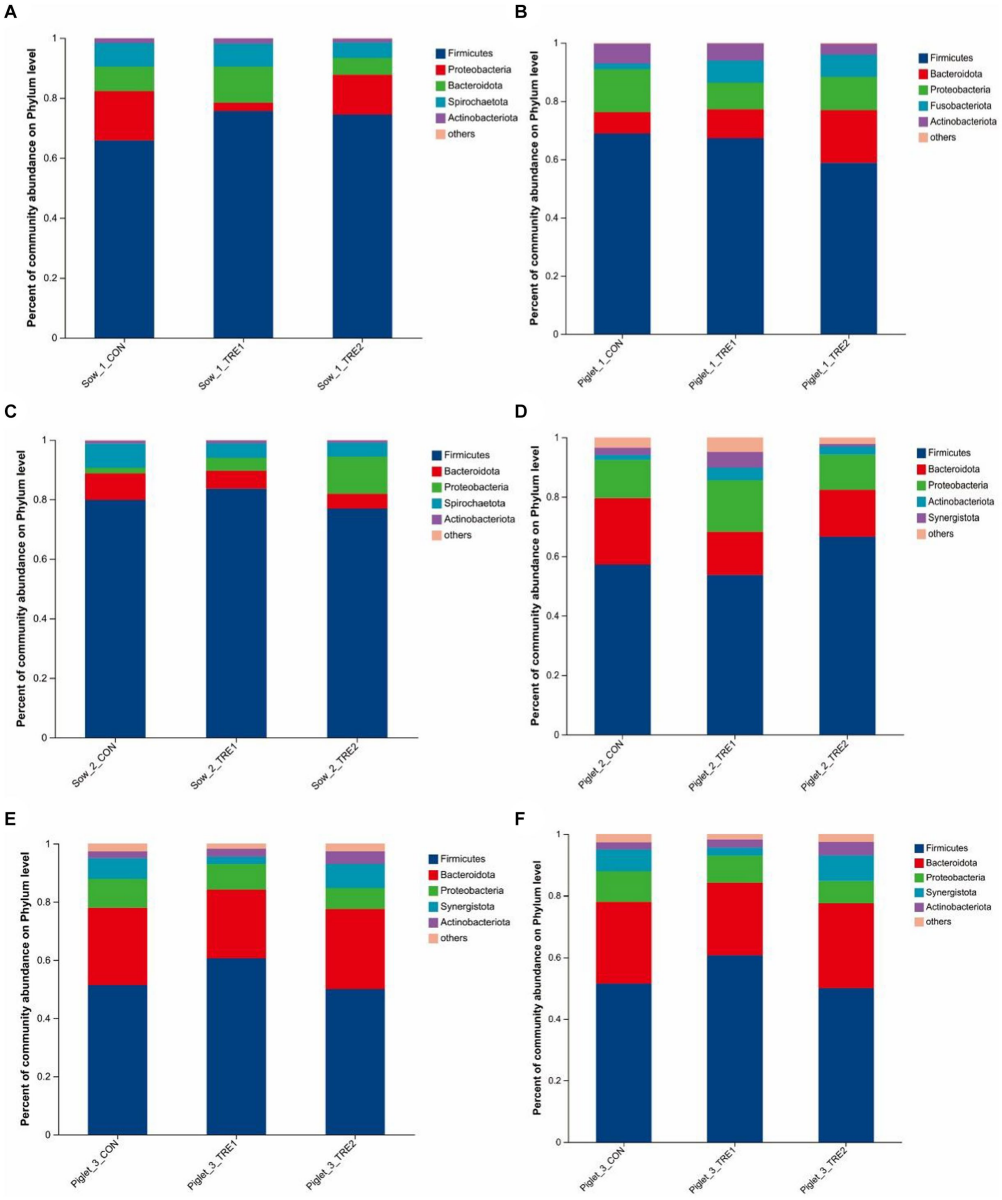
Fecal microbiota composition in sows and piglets of different groups at the phylum level. Relative abundance of sow fecal phylum microbiota in the control group (CON), treatment group 1 (TRE1), and treatment group 2 (TRE2) on the first day after delivery **(A)**, the seventh day after delivery **(C)**, and the 21st day after delivery **(E)**. Relative abundance of suckling piglet fecal microbiota in the control group (CON), treatment group 1 (TRE1), and treatment group 2 (TRE2) on the first day after delivery **(B)**, the seventh day after delivery **(D)**, and the 21st day after delivery **(F)**. *n* = 6 for each group.

The fecal microbial composition at the phylum level of sows and suckling piglets in different experimental groups is shown in Figure 3. As can be seen from Figures 3A,C,E, the top five dominant bacteria in sow feces were *Firmicutes*, *Proteobacteria*, *Bacteroidota*, *Spirochaeta*, and *Actinobacteria*. Compared with CON, the proportion of *Firmicutes* in TRE1 and TRE2 increased by 9.81 and 8.60% on the first day after delivery, respectively. On the seventh day after delivery, the proportions of *Firmicutes* in TRE1 increased by 3.81%, while that of *Proteobacteria* in TRE2 increased by 10.65%. On the 21st day after delivery, the proportion of *Firmicutes* in TRE1 decreased by 2.93%, while that of *Spirochaeta* in TRE2 increased by 2.04%. The dominant bacterial phyla in sows were relatively stable at different stages after delivery. Compared with rice husk meal, BBF1 supplementation mainly changed the proportion of *Firmicutes*.

According to Figures 3B,D,F, the top dominant bacteria in the feces of suckling piglets were *Firmicutes*, *Bacteroidota*, *Proteobacteria*, *Fusobacteriota*, *Actinobact eriota*, *Synergistota*, and *Spirochaeta*. *Synergistota* was added as the dominant bacteria to the feces of suckling piglets on the seventh day after delivery, and *Spirochaeta* was added as the dominant bacteria on the 21st day after delivery. Compared with CON, on the first day after delivery, the proportion of fecal *Fusobacteriota* of piglets in TRE1 increased by 5.71%, and *Bacteroides* in TRE2 increased by 10.75%. On the seventh day after delivery, the proportion of *Proteobacteria* in TRE1 increased by 4.53%, and *Firmicutes* in TRE2 increased by 9.40%. On the 21st day after delivery, the proportion of *Firmicutes* in TRE1 increased by 9.22%, and proportion of *Proteobacteria* in TRE2 decreased by 2.71%.

**Figure 4:**
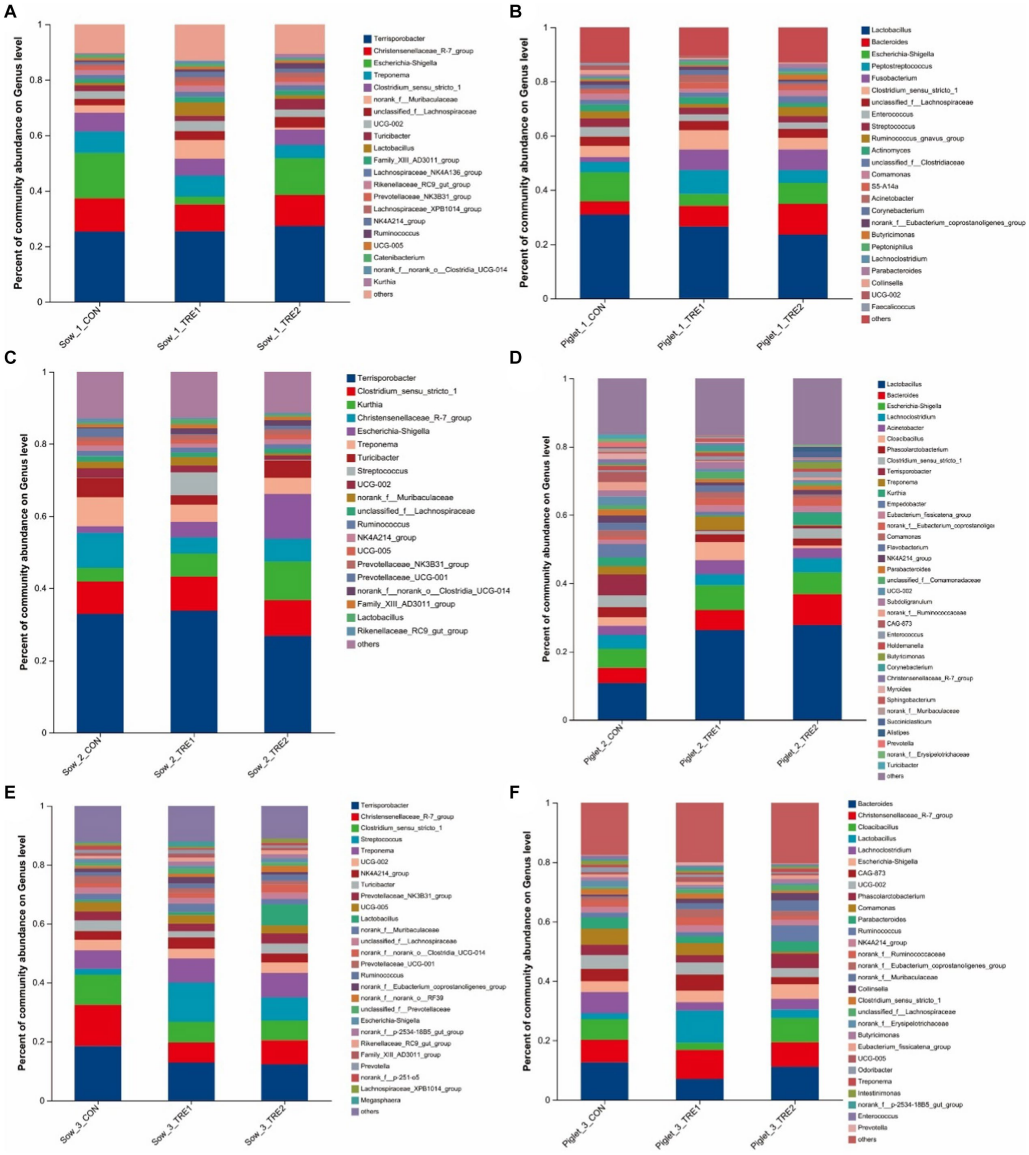
Fecal microbiota composition in sows and piglets of different groups at the genus level. Relative abundance of sow fecal genera microbiota in the control group (CON), treatment group 1 (TRE1), and treatment group 2 (TRE2) on the first day after delivery **(A)**, the seventh day after delivery **(C)**, and the 21st day after delivery **(E)**. Relative abundance of suckling piglet fecal microbiota in the control group (CON), treatment group 1 (TRE1), and treatment group 2 (TRE2) on the first day after delivery **(B)**, the seventh day after delivery **(D)**, and the 21st day after delivery **(F)**. *n* = 6 for each group.

The fecal microbial composition at the genus level of sows and suckling piglets in different experimental groups is shown in Figure 4. As seen from Figure 4A, on the first day after delivery, there were 21 dominant bacterial genera in the feces of sows of all three groups, including *Terrisporobacter*, *Christensenaceae R-7 group*, *Escherichia-Shigella*, *Treponema*, *Clostridium_sensu_stricto_1*, and so on. The proportions of dominant bacteria in CON, TRE1, and TRE2 were 89.69, 87.00, and 89.38%, respectively. As seen in Figure 4B, there were 24 dominant bacterial genera in the feces of suckling piglets, accounting for 86.96, 89.44, and 87.10% in CON, TRE1, and TRE2, respectively. Different from the dominant bacteria composition in sow feces, there were 19 dominant bacterial genera unique to suckling piglets, including *Bacteroides*, *Peptostreptococcus*, *Fusobaterium*, *Enterococcus*, *Streptococcus*, and so on.

As seen from Figure 4C, on the seventh day after delivery, there were 20 dominant bacterial genera in the feces of sows of all three groups, including *Terrisporobacter*, *Clostridium_sensu_stricto_1*, *Kurthia*, *Christensenaceae R-7 group*, *Escherichia–Shigella*, and so on. The proportions of dominant bacteria in CON, TRE1, and TRE2 were 87.11, 87.27, and 88.65%, respectively. According to Figure 4D, there were 36 dominant bacterial genera in the feces of suckling piglets, accounting for 83.60, 83.32, and 80.57% in CON, TRE1, and TRE2, respectively. Different from the dominant bacteria composition in sow feces, there were 25 dominant bacterial genera unique to suckling piglets, including *Bacteroides*, *Lachnoclostridium*, *Acinetobacter*, *Cloacibacillus*, *Phascolarctobacterium*, and so on.

As seen from Figure 4E, on the 21st day after delivery, there were 27 dominant bacterial genera in the feces of sows of all three groups, including *Terrisporobacter*, *Christensenellaceae_R-7_group*, *Clostridium_sensu_stricto_1*, *Streptococcus*, *Treponema*, and so on. The dominant bacterial genera in CON, TRE1, and TRE2 accounted for 87.78, 87.83, and 88.87%, respectively. As seen in Figure 4F, there were 29 dominant bacterial genera in the feces of suckling piglets, accounting for 82.46, 79.97, and 79.55% in CON, TRE1, and TRE2, respectively. Different from the dominant bacteria composition in sow feces, there were 15 dominant bacterial genera unique to suckling piglets, including *Bacteroides*, *Cloacibacillus*, *Lachnoclostridium*, CAG-873, *Phascolarctobacterium*, and so on.

**Figure 5:**
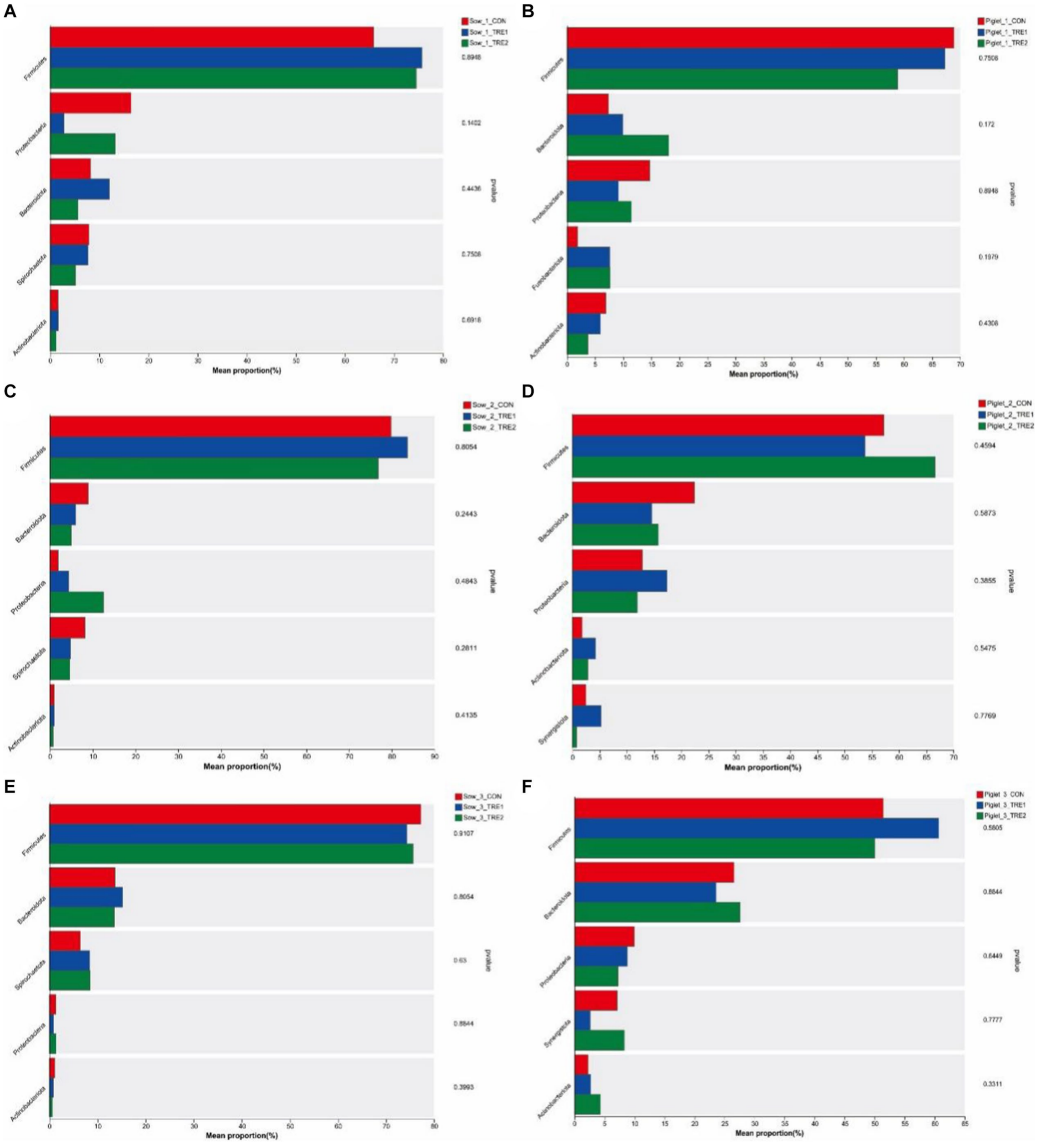
Differences in the fecal microbiota of sows and piglets at the phylum level in different experimental groups. Fecal phylum microbiota differed in sows among the control group (CON), treatment group 1 (TRE1), and treatment group 2 (TRE2) on the first day after delivery **(A)**, the seventh day after delivery **(C)**, and the 21st day after delivery **(E)**. Fecal phylum microbiota differed in suckling piglets among the control group (CON), treatment group 1 (TRE1), and treatment group 2 (TRE2) on the first day after delivery **(B)**, the seventh day after delivery **(D)**, and the 21st day after delivery **(F)**. *n* = 6 for each group.

The differences of top five fecal dominant bacterial phyla of sows and suckling piglets among different groups are shown in Figure 5. There was no significant difference in the abundance of dominant bacteria at the phylum level for sows and suckling piglets among CON, TRE1, and TRE2 on the 1st, 7th, and 21st days after delivery (*p* > 0.05).

**Figure 6:**
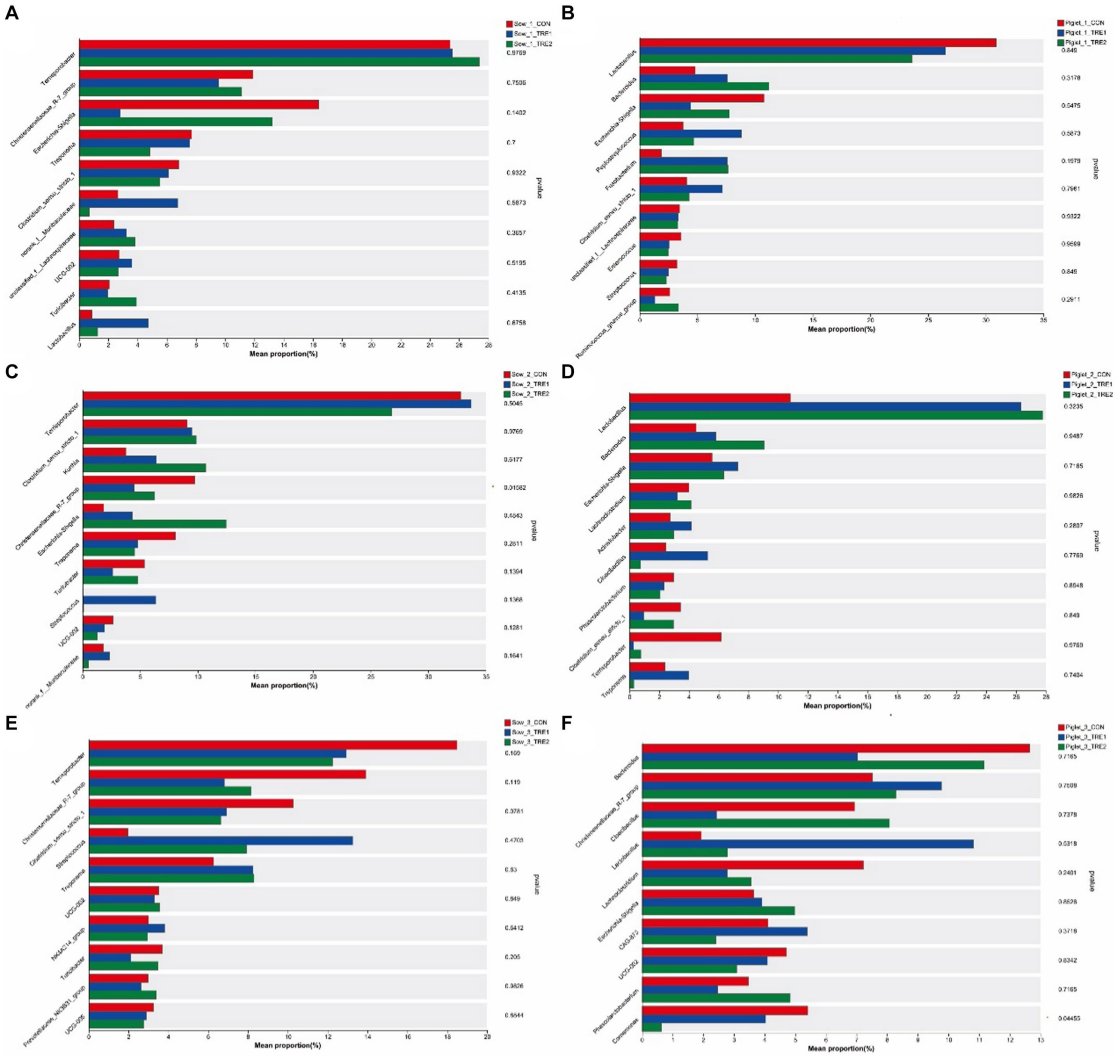
Differences in the fecal microbiota of sows and piglets at the genus level in different experimental groups. Fecal genera microbiota differed in sows among the control group (CON), treatment group 1 (TRE1), and treatment group 2 (TRE2) on the first day after delivery **(A)**, the seventh day after delivery **(C)**, and the 21st day after delivery **(E)**. Fecal genera microbiota differed in suckling piglets among the control group (CON), treatment group 1 (TRE1), and treatment group 2 (TRE2) on the first day after delivery **(B)**, the seventh day after delivery **(D)**, and the 21st day after delivery **(F)**. *n* = 6 for each group.

**Figure 7:**
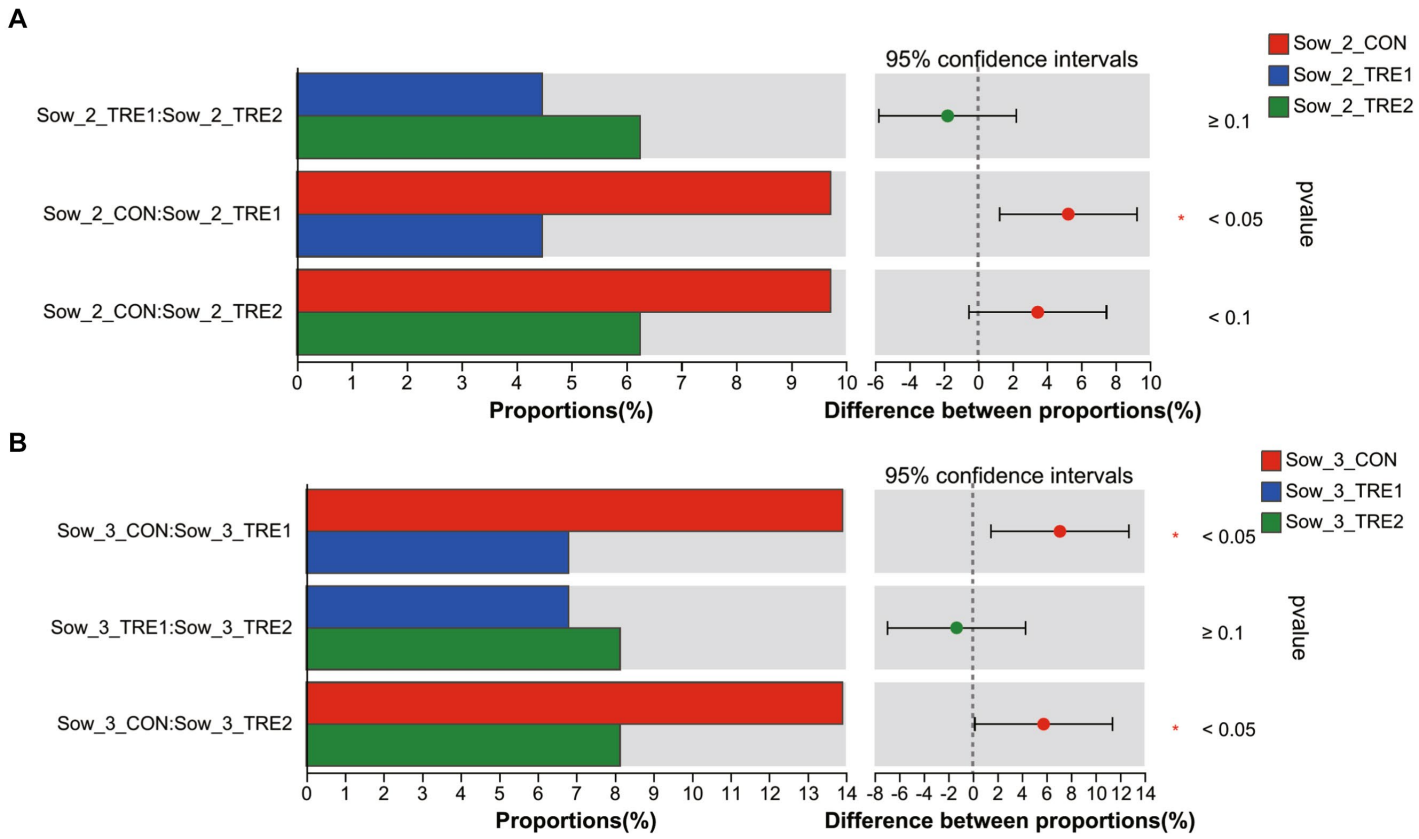
Differences in sows fecal genera *Christensenellaceae_R-7_group* between different experimental groups. Fecal bacterial genera differed in sows between CON and TRE1, CON and TRE2, and TRE1 and TRE2 on the seventh day after delivery **(A)** and the 21st day after delivery **(B)**. *n* = 6 for each group.

The differences of top 10 fecal dominant bacterial genera of sows and suckling piglets among different groups are shown in Figure 6. According to Figures 6A,B, there were no significant differences in the abundance of dominant bacteria at the genus level for sows and suckling piglets in CON, TRE1, and TRE2 on the first day after delivery (*p* > 0.05). As shown in Figures 6C,D, there were significant differences in fecal *Christensenellaceae_R-7_group* in sows of CON, TRE1, and TRE2 on the seventh day after delivery (*p* < 0.05), and compared with CON, genera *Christensenellaceae_R-7_group* was significantly decreased by BBF1 supplementation (*p* < 0.05), while BBF2 tended to decrease *Christensenellaceae_R-7_group* (*p* < 0.10) (Figure 7A). However, there were no significant differences in the dominant bacterial genera in the feces of suckling piglets among these groups (*p* > 0.05). As shown in Figures 6D,E, a trend difference in the sows feces of CON, TRE1, and TRE2 was found in *Clostridium_sensu_stricto_1* on the 21st day after delivery (*p* < 0.10), while there was no significant difference in the genera *Clostridium_sensu_stricto_1* between CON and TRE1 or between CON and TRE2 (*p* > 0.05). Compared with CON, the genera *Christensenellaceae_R-7_group* was significantly decreased by BBF1 and BBF2 supplementation in the feces of sows (*p* < 0.05) (Figure 7B). There was a significant difference in *Comamonas* of suckling piglets by dietary fiber (*p* < 0.05), while there was no significant difference between CON and TRE1 or between CON and TRE2 (*p* > 0.05).

### Correlation between fecal microorganisms and growth performance of suckling piglets

**Figure 8:**
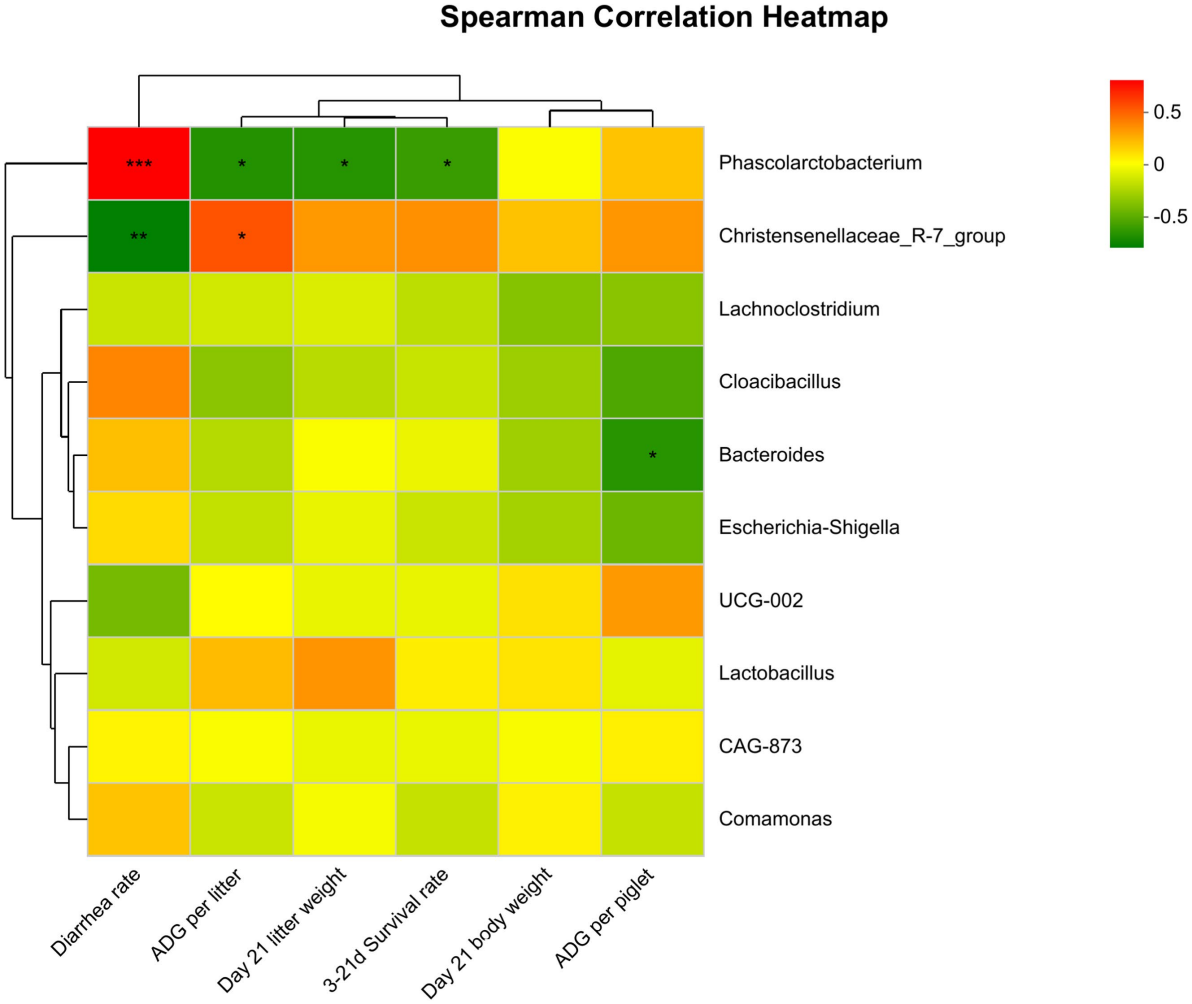
Spearman correlation analysis between differential genera and growth performance of piglets. Significant correlations are noted by *0.01 < *p* ≤ 0.05, **0.001 < *p* ≤ 0.01, ***p ≤ 0.001.

As shown in Figure 8, Spearman correlation analysis found that the abundance of *Phascolarctobacterium* in the feces of suckling piglets on the 21st day was significantly positively correlated with diarrhea rate (*p* ≤ 0.001). It was negatively correlated with 21-day litter weight, average daily gain, and 3- to 21-day survival rate (0.01 < *p* ≤ 0.05). There was a significant negative correlation between the abundance of *Bacteroides* and the ADG of piglets (0.01 < *p* ≤ 0.05). In contrast, the abundance of *Christensenellaceae_R-7_group* was significantly negatively correlated with diarrhea rate (0.001 < *p* ≤ 0.01) and positively correlated with litter ADG (0.01 < *p* ≤ 0.05).

## Discussion

### Effects of maternal bamboo fiber supplementation on reproductive performance of sows and growth performance of piglets

It has been demonstrated that insufficient feed intake during lactation led to a deficiency in the sow’s milk production and a lack of overall nutrients required for piglet growth (Kim and Wu, 2004), which may limit the growth and development of piglets (He et al., 2017). Sows fed a high-soluble fiber and high-insoluble fiber diet had a significantly greater ADFI and lost less BW during lactation (Renteria-Flores et al., 2008). In this study, the ADFI of sows supplemented with dietary BBF1 was significantly higher than that with rice husk meal, while there was no significant difference between the BBF2 and CON groups. This suggests that the feed intake of lactating sows may be affected by fiber characteristics in addition to fiber level, and soluble fiber may affect the gastrointestinal chyme velocity and thus reduce feed intake.

Since the necessary nutrition for the fetus and newborn piglets comes mainly from the sows (Frese et al., 2015; Grześkowiak et al., 2022), this sow-piglet association influenced the performance of their offspring piglets. The backfat thickness change of sows during gestation did not differ among the groups in this study, which was consistent with earlier research showing that dietary fiber supplementation had no effect on body weight gain of sows during gestation when energy was similar, regardless of fiber source (Guillemet et al., 2007). This means that sows in the high feed intake group are more conducive to the growth of their offspring. In our study, dietary supplementation of 2% BBF1 in sows had a significant effect on ADG per litter at pre-weaning 11–21 days and 3–21 days, increasing by 50.68 and 51.70%, 31.61 and 34.71%, respectively, compared with the control and TRE2 groups. The addition of BBF1 also improved BW per litter at 21-day weaning and the survival rate of 3- to 21-day-old piglets. Similarly, maternal sugar beet pulp supplementation was more effective than wheat bran in improving sow ADFI and enhancing the growth performance of their piglets (Shang et al., 2019). The above studies indicate that it is feasible to increase the ADFI of lactating sows and improve the growth performance of their piglets by adding appropriate bamboo powder fiber.

### Effects of maternal bamboo fiber supplementation on serum biochemical indices of sows

Dietary fiber regulates serum fat metabolism. Our previous study showed that the serum triglyceride concentration and total cholesterol decreased significantly with the increase of bamboo powder from 0 g/day, 30 g/day, and 60 g/day in the diets of perinatal sows (Dai et al., 2023). Another study in pregnant sows found that dietary supplementation with beet meal or bran significantly reduced serum total cholesterol in sows at 110 days of gestation (Shang et al., 2021). This result was also confirmed in an experiment on growing pigs. Dietary supplementation of fibrous raw materials (such as flaxseed meal and oat hulls) stimulated the excretion of bile acids and neutral sterols, thereby significantly reducing fat digestibility and serum cholesterol levels (Ndou et al., 2017). In this study, it was found that the serum total cholesterol and total triglyceride contents in TRE1 supplemented with BBF1 on the third day after delivery were higher than those in CON supplemented with rice husk meal, and the contents of total triglyceride reached a significant level on the 21st day after delivery, and the contents of total cholesterol were higher than those in CON. This suggests that bamboo fiber may be more beneficial to promote the lipid metabolism of sows than rice husk meal, which may also be the reason why it is more beneficial to improve the weight gain of piglets.

Interestingly, we found that the fibers in the above trial can be divided into two categories: soluble fibers, including beet meal and flaxseed meal; and insoluble fibers, including bran, oat shell, feeding bamboo powder, etc. However, they all regulated the lipid metabolism of pigs, which indicated that the serum total cholesterol and triglyceride levels are mainly affected by the fiber level, and the fiber component may not be the main influencing factor. However, another study found that different types of fiber showed inconsistent regulatory effects on the serum triglycerides of sows at different stages (Weng, 2020). Therefore, it is necessary to further study the application of different bamboo fibers in different physiological stages and their influence on the fat digestibility and milk fat content of sows.

Serum total protein and serum urea nitrogen levels can reflect the efficiency of protein utilization in pigs (Kohn et al., 2005). Changes in the serum urea nitrogen concentration of sows at different physiological stages may be the result of changes in diet or body protein breakdown (Muller et al., 2022). It was shown that serum total protein concentration increased significantly with the increase in dietary fiber supplementation level when adding citrus pulp, maize cob, and lucerne hay, which had a negative effect on production performance (Bakare et al., 2016). Our previous study found that supplemental feeding of 30 g/day or 60 g/day bamboo powder in the diet of sows during the perinatal period had no significant effects on serum total protein and urea nitrogen of sows (Dai et al., 2023), and similar results were obtained in this experiment. The above studies indicated that 2% BBF supplementation had no negative effects on the protein metabolism of sows during gestation and lactation. Supplemental feeding of bamboo powder may have similar protein metabolism and utilization as rice husk meal, and it might also be related to the physiological stage of sows.

### Effects of maternal bamboo fiber supplementation on fecal microorganisms of sows and suckling piglets

Fiber nutrition of sows plays an important role in regulating the intestinal microbial composition and health of their offspring piglets. The gut microbial diversity was the main response to dietary fiber intake (Koh et al., 2016). It was found that dietary GCW (a compound SDF raw material) reduced gut microbial diversity and the relative abundance of bile salt hydrolase gene-encoding bacteria, *Lactobacillus* and *Bacteroides*, in sows on day 109 of gestation (Wu et al., 2021). Another study found that adding wheat bran, soybean hull, and sugar beet pulp to the high dietary fiber diet of sows significantly increased microbial diversity and abundance of *Acidobacteria* and *Bacteroidetes* in the colonic chyme of their piglets (He et al., 2020). Our previous study found that the supplemental feeding of bamboo powder in perinatal sows significantly reduced the fecal microbial diversity index of sows, while it tended to increase the fecal microbial diversity index of their piglets (Dai et al., 2023). These studies indicate that maternal dietary fiber supplementation has affected microbial diversity in their suckling piglets, which confirms the existence of “sow-piglet” association. Similarly, in this study, it has been found that bamboo fiber supplementation for sows tended to increase the fecal Sobs index of suckling piglets on the 21st day after delivery. These results suggested that adding bamboo fiber (a natural IDF raw material) to the high dietary fiber diet of sows might help increase the microbial diversity of offspring piglets.

The fiber of rice husk meal and feeding bamboo powder was mainly composed of IDF, and IDF had a slow fermentation speed and produced less short-chain fatty acid (SCFA) than soluble fiber, thereby reducing the richness of the gut microbiota (Jha and Berrocoso, 2015). Another study found that there were no differences in the fecal microbial diversity index among the sows treated with lignocellulose, resistant starch, and konjac flour (Lu et al., 2022). Similarly, even sugar beet pulp (SBP) and oat bran (OB) had a similar ratio of SDF to IDF, while the predicted acetate, total SCFA production, and absorption in the SBP group were higher than those of the OB group (Bai et al., 2022). In this study, it was shown that the fecal microbial diversity index of sows and piglets in the group with BBF1 was higher than that with rice husk meal on days 7 and 21 of lactation, but the addition of bamboo fiber polymer material (SDF) on the basis of BBF1 did not further improve the microbial diversity. These results indicated that the effects of SDF and ISF on microbial diversity were not consistent, and it might be more important to select appropriate dietary fiber sources.

*Firmicutes* was the dominant bacteria in the fecal flora of healthy sows, followed by *Proteobacteria* and *Bacteroidetes* (Zhang et al., 2021). This study also found that the top five dominant bacteria in sow feces were *Firmicutes*, *Proteobacteria*, *Bacteroidota*, *Spirochaetota*, and *Actinobacteriota*. *Firmicutes* played a significant role in the degradation of complex plant carbohydrates and the key metabolic conversions within the intestinal community, such as the major butyrate-producing species (Flint et al., 2012). The fecal dominant genera varied as piglets aged, and transitions toward a mature gut microbiota enriched with fiber-degrading bacteria were mostly completed upon weaning in piglets with better growth (Mahmud et al., 2023). This study found that the top five dominant bacteria in sows feces were relatively stable at different stages after delivery, while the top five dominant bacteria of piglets had evolved and added *Synergistota* on day 7 after delivery and *Spirochaeta* on day 21 after delivery. These results indicated that the gut microbiota of piglets might be susceptible to environmental changes after birth.

It has been proven that many fiber-rich materials affect the intestinal flora composition of sows. A study found that stevia-residue supplementation promoted the relative abundance of fecal beneficial bacteria of pregnant sows, such as *g__Lachnospiraceae_XPB1014_group* and *g__Christensenellaceae_R-7_group*, and reduced the relative abundance of harmful bacteria, such as *Treponema_2* (Yu et al., 2020). Another study found that the addition of 5% *Pennisetum purpureum* during late pregnancy of sows significantly increased the relative abundance of *Bacteroidetes*, *Actinobacteria*, and *Prevotellaceae_UCG_001* and decreased the abundance of *Escherichia_Shigella* (Huang et al., 2021). In recent years, bamboo feed resources have been developed and applied to pig production. A study found that the addition of 12% fermented bamboo shoot processing waste had beneficial effects on the gut microbiota of weaned piglets and significantly reduced the taxon feature number and the relative abundance of *Tenericutes* in the cecum (Huang J. et al., 2022; Huang Y. et al., 2022). Another study found that the intake of fermented bamboo fiber by sows promoted the enrichment of beneficial genera such as *Lachnospira*, *Lachnospiracea_XPB1014_Group*, and *Roseburia* and reduced the relative abundance of harmful bacteria such as *Fusobacterium*, *Sutterellaceae*, and *Sutterella*, while significantly increasing the relative abundance of beneficial bacteria *Alistipes* and *Lachnoclostridium* and decreasing the pathogenic bacteria *Trueperella* of piglets (Sun et al., 2023). In this study, it was found that bamboo fiber supplementation decreased the fecal genera *Christensenellaceae_R-7_group* of sows with no significant effect on their suckling piglet. These results indicated that bamboo fiber and rice husk meal had different regulatory effects on the microbial composition of sows, although both of them were IDF fibers.

### Associations between fecal microbes and growth performance of suckling piglets

It has been shown that the gut microbiota changes dramatically across different reproductive stages (Cheng et al., 2018). Fiber ingestion during the suckling period is helpful for gut development and probiotic colonization. It has been shown that xylooligosaccharide ingestion in suckling piglets improved growth performance and feed efficiency after weaning by increasing the fermentation capacity of the microbiota and fiber-degrading enzyme secretion (Bai et al., 2021a). According to the difference in fecal microbial composition of piglets among different fiber treatment groups, this study focused on analyzing the correlation between fecal microbial composition and growth performance of piglets on day 21 after delivery. The diarrhea rate was associated with increases in the relative abundance of *Prevotella*, while the increased relative abundance of *Prevotella* was correlated with a reduction in *Escherichia coli* and the majority of beneficial bacteria belonging to the *Firmicutes* phylum in piglets (Yang et al., 2017). Another study showed that the abundance of *norank_f__Muribaculaceae*, *Christensenellaceae_R-7_group*, *Enterococcus*, and *Romboutsia* had a positive connection with almost all barrier function genes of neonatal piglets on day 21 (Wu et al., 2020). It was shown that butyrate production could be improved by inhibiting the proliferation of *Lachnospiraceae_XPB_1014_group* and *Bacteroides*, which had a lack of potential to secrete β-xylosidase (Bai et al., 2021b). This study also found that there was a significant negative correlation between the abundance of *Bacteroides* and ADG in piglets, while the abundance of *Christensenellaceae_R-7_group* was significantly negatively correlated with diarrhea rate and positively correlated with litter ADG. This suggests that maternal bamboo fiber supplementation can improve the growth performance of piglets by promoting the abundance of beneficial flora.

## Conclusion

It was shown that maternal bamboo fiber supplementation during perinatal and lactation increased the feed intake of sows during lactation, litter weight gain, weaning litter weight, and serum triglyceride of piglets. This beneficial effect of bamboo fiber was associated with the improvement of diversity and structure of fecal microbiota in the piglets.

## Data availability statement

The data presented in this study are deposited in the NCBI repository, accession number PRJNA1119022.

## Ethics statement

The animal study was reviewed by Animal Care and Use Committee of Leshan Normal University (Certification No. 4151010649), China, and conducted in accordance with the approved protocol (No. LAC2023006). Written informed consent was obtained from the owners for the participation of their animals in this study.

## Author contributions

FD: Conceptualization, Data curation, Funding acquisition, Methodology, Project administration, Writing – original draft, Writing – review & editing. TL: Conceptualization, Investigation, Software, Supervision, Writing – review & editing. MJ: Data curation, Formal analysis, Investigation, Methodology, Resources, Software, Writing – original draft. XH: Data curation, Methodology, Writing – original draft. LW: Software, Supervision, Writing – review & editing. JM: Data curation, Formal analysis, Methodology, Writing – original draft. HY: Data curation, Formal analysis, Methodology, Writing – original draft. XF: Data curation, Formal analysis, Methodology, Writing – original draft. XN: Writing – review & editing. JZ: Writing – review & editing.

## Footnotes

^1^https://github.com/OpenGene/fastp, version 0.19.6.

^2^http://www.cbcb.umd.edu/software/flash, version 1.2.11.

^3^http://drive5.com/uparse/, version 7.1.

^4^http://rdp.cme.msu.edu/, version 2.11.

^5^ http://www.arb-silva.de

^6^ https://cloud.majorbio.com

^7^ http://www.mothur.org/wiki/Calculators
